# Essential Points in the After-Treatment of Surgical Cases1Read at Rochester Pathological Society meeting, February 13, 1897.

**Published:** 1897-06

**Authors:** S. L. Elsner

**Affiliations:** Rochester, N. Y. 83 North St. Paul Street


### ESSENTIAL POINTS IN THE AFTER-TREATMENT OF SURGICAL CASES.[1]

By S. L. ELSNER, M. D., Rochester, N. Y.

KNOWING that the society had had a most interesting series of papers on special subjects, I will try to make a more general subject interest you this evening, and thereby hope to gain many points in the after-treatment of our surgical cases. Medical journals are constantly giving us articles on operations for abdominal and pelvic disease, and usually the surgeon spares himself no labor, time or expense in compiling statistics, in demonstrating his technique, and in the reporting of his histories. In view of all this, is it not deplorable that our most experienced workers fail to give the details of the after-treatment in this class of cases ?

Aside from the subject of drainage very little has been said or written. We are all aware that the patient's comfort and safety depend principally upon the kind of operation and the technique employed, but we are just as positive that they are greatly influenced by the treatment during, and that immediately follow-

1. Read at Rochester Pathological Society meeting, February 13, 1897.

ing, the surgical procedure. The operator has been far more successful since he has adopted a systematic and well-organised plan in the preparation of his patient. How greatly would we appreciate the advantage of such fixed rules in the after-treatment of abdominal section. Of course, details must vary with the necessities of each individual case, yet it shall be my purpose to collect and arrange such principles as may seem best suited for general adoption.

### PROPHYLACTIC MEASURES.

Let us first consider a few prophylactic measures that may lessen the necessity for after-treatment; we will take it for granted that the patient is prepared as is now our universal custom. The anesthetic should be chloroform, unless there be a marked contra-indication for its use. We thus avoid the unpleasant inhalation of ether, the struggling, and what is most important, lessen the chances of nausea and vomiting afterward. The amount of anesthetic, whether chloroform or ether, should be as small as will keep the patient relaxed. The patient should be handled as gently as if conscious and any unnecessarily strained position of the body avoided. Hospital attendants are very apt to be neglectful of these and the patient often wonders how it is that the limbs, body, face, tongue and jaw are so sore and lame. Hypodermics, when necessary, should be given most carefully and superficial punctures should be avoided. When, because of the magnitude of an operation or the previous mental or physical condition of the patient, you expect trouble from shock, it will be far better to anticipate the same by a few hypodermics of strychnine, and perhaps a few drops of amyl nitrate in the anesthetic, than to wait until a rapid and feeble pulse, with a clammy surface, demand urgent treatment. The temperature of the room should be well regulated and all possible draughts avoided. The body should be well surrounded by warm blankets and sheets, and as small a portion of the abdomen exposed as is consistent with doing good work. In operating rooms supplied with the Edison current the electric heating pad is quite an advantage. It can be placed under or over the patient and the temperature accurately regulated, thus giving a very convenient appliance for keeping the body warm. Do not try to work through an inch or an inch and a half incision when you find that more room would save time and also avoid undue violence to the abdominal organs. When in doubt as to what you feel, a larger incision will permit of more light being thrown in and one can see what is being done.

When it is necessary to protect part of the intestines outside of the wound, moist hot towels to cover them are less liable to cause abrasions to their peritoneal coat than are hot dry towels or sponges. Should it be advisable to flush the abdominal cavity, the fluid used should be at least 110° F. and the quantity large. The irrigation from a small tip is not efficient; a large tube with a funnel attached should be used, or the water poured directly from a pitcher. I have often seen patients with a very poor pulse at this stage of an operation react most kindly after a flushing of the peritoneum with a hot saline solution. All oozing should be checked and the cavity, in cases not flushed out, should be left as dry as possible.

Time is an important feature. The most successful operator is usually the one who is thorough and yet quick. After the peritoneal cavity is shut off, any time lost in carefully adapting the fascia and skin does not materially increase the risk.

### POST OPERATIVE CARE.

Having considered these prophylactic measures, let us advance to the means of caring for the patient after the operation. On leaving the operating table it is well to put the patient in a bed previously warmed and supplied with hot cans. The recovery rooms should not be much cooler for a time than was the operating room, and the halls leading from one to the other of these rooms should be free from draughts. The nurse that takes charge of the patient should, if possible, be present at the operation, so that she is acquainted with the location of wounds, drainage-tubes and dressings, and consequently better able to make her patient comfortable when a change of position is required. If the patient is not shocked, the first symptom that may require treatment is the nausea, with or without vomiting. This usually ceases within a few hours, particularly if chloroform was administered. If it continues, however, it becomes a most annoying thing to treat, and a most distressing feeling for the patient with an abdominal or pelvic wound to endure. By the treatment for its relief one is often baffled. My plan is to keep the stomach at rest by withholding food or drink. If thirst is complained of, I give from six to ten ounces of normal salt solution per rectum every three or four hours, alternating this with nutritive enemata should feeding become necessary, and occasionally allow washing of the mouth with cold water ; thus you feed and stimulate your patient and still avoid any unnecessary vomiting and retching. If this does not control the vomiting, you will find that a few rectal injections of from fifteen to twenty-five grains of chloral hydrate, diluted in two ounces of either warm water or milk, repeated at three-hour intervals, will soon bring about the desired result. It is not at all uncommon for my cases to go seventy-two hours without food or drink. I do this, not because of the presence of nausea or vomiting, but because I make it a fast rule to have nothing enter the stomach until gas or fecal matter has been expelled. I do believe that this avoids distress from gas, peritoneal inflammation and subsequent adhesions, with the possibility of intestinal obstruction. Morphia or other opiates should be withheld, unless severe pain demand them ; their use only increases the vomiting and nausea, paralyses the intestinal coats and makes it all the harder to get an action of the bowels.

Absolute rest of mind as well as body is essential. The room should be pleasant, commodious and well aired, and when possible the patient should not be left without a constant attendant for the first three days. A position on the back should be maintained for a few days. Every movement causes pain and increases the nausea of an already irritable stomach. By carelessness of the nurse or patient, in changing position, a ligature may slip, a glass drain may break, or the edges of a wound may be disturbed by the moving of the dressings. Often a patient lying on the back will demand a change when, if the nurse simply attended to the smoothing of the sheet and night-dress, or a change in the position of the limbs, her patient would at once be comforted. Occasionally a hand moistened with alcohol worked under the bandage and applied to the back, or the same applied to the forehead, temples, cheek and arms, brings with every stroke of a willingly applied palm solace and comfort to the suffering individual.

All visitors, as well as their floral offerings, should be refused for the first forty-eight or seventy-two hours, and for the first week thereafter only one or two of the nearest relatives should be allowed to visit the patient for a few moments each day. The bowels should receive attention at as early a date as is consistent with the kind of operation performed. In all cases, except those in which the intestines themselves have been involved, either in the way of anastamosis or suture, we should begin to move them at the end of twenty-four hours, sooner if there be any distention. The bowels and pulse are the best guide to the progress of the case. If the pulse is over 110 with a tendency to become more rapid, and the abdomen is distended, immediate purgation is indicated, and it is astounding what relief a good movement brings. The rapid pulse falls, distention is over, the anxious expression of the face changes to one of contentment and comfort. If the distention and rapid pulse continue, however, and you are unable to open the intestinal tract, you may make up your mind that the case will not terminate favorably. Fourteen to sixteen hours after the operation, it is well to start the patient on grain doses of calomel every hour until six to ten grains have been taken. Follow this by fluid citrate of magnesia, two ounces every half to one hour until eight to sixteen ounces are taken. In the meantime it is well to make a high injection of turpentine, one ounce ; magnes sulphas, two ounces ; glycerine, four ounces ; and soap water to make two quarts. You will find the plan of using salt solution to quench thirst, in the way spoken of, greatly aiding the result; for it is not uncommon to have gas pass the rectal tube while trying to introduce the same high up for these injections, and often, too, the bowels will move while the nurse is trying to have a nutritive enema retained.

It is fair to prognosticate that if the third day has passed, after an abdominal or pelvic operation, and the bowels have moved well, that your patient is on the road to a speedy convalescence.

The bladder is our next consideration. No matter how aseptic your nurse and her catheter, in the majority of cases you will find that frequent catheterisation will soon produce an irritable, if not an inflammatory, condition in this viscus, so that one cannot be too careful. Always use a glass catheter. I have twice seen the nurse use a bichloride dossil, and wash the vulva from behind forward, thereby bringing all the possible available infection from rectum to the urethra. Do not leave an order with the nurse to catheterise every six hours, as is the usual method, but rather say no longer than twelve hours, and sooner if symptoms call for it.

I have often found that allowing the bladder to become a little distended enabled the patient to pass the urine voluntarily, and unless there be some contraindication for this slight voluntary effort, it will often avoid the usual twenty-four or forty-eight hours of catheterisation and the unpleasant bladder symptoms following. At the slightest suspicion of cystitis, irrigate the bladder with some bland antiseptic and repeat if necessary. After the stomach is quiet and the bowels have moved, the question of how best to nourish these patients arises. To be slow and sure with this stage of advancement is the best plan. Begin with half-ounce doses of milk, mineral water or broth every half to one hour, and gradually increase the amount to four to eight ounces, and lengthen the intervals to two hours. After two days of such feeding and all goes well, the patient is ready for any light food she may fancy. Never force any particular kind of nourishment, but rely principally upon that which your patient finds most palatable and best borne. After the patient begins to take food in this way, do not limit the amount of fluid or order its administration at just such hours. You will observe, if you will take the trouble, that many of these cases feel that they are about starved, and they seem to at times need the food at very frequent intervals, and almost instantly after the desire comes on. I presume that this craving is due to the fact that the bloodvessels, not having been able to satisfy their demands for fluids from the stomach, draw upon every conceivable source for their need, and thus actually sponge up every bit of fluid they can reach. This very sponging up only strengthens my belief that withholding food in these cases, as I have stated, helps to prevent inflammatory trouble. Often, were germs to meet with say a little serum or blood that had been left in the peritoneal cavity, that had not been subject to nature's method of sponging, they would undoubtedly find a most perfect soil for their immediate culture and prolification.

I have said very little about flatulency, excepting as I detailed the manner of opening the bowels. What I will say further is only to condemn all methods of intestinal puncture, opening of the incision and cutting into the intestine, washing the stomach, and the like. Passing a large rectal tube, ten or twelve inches, will give as much relief as any of the above, and if the annoying symptom is due to a paralysis of the bowels with septic peritonitis, nothing short of death gives any relief.

The treatment of surgical shock is fairly well understood. I would suggest one thing, however, and that is, never despair of your chance for success as long as the patient makes the slightest effort at breathing. The methods most successful are heat applied about the body, strychnine hypodermically, and transfusion or subcutaneous injections of the normal saline solution. It is surprising how some of these patients emerge even from what seems a hopeless condition, when these means for reaction are diligently persisted in.

Hemorrhage has but one treatment. Find its source, and ligate, or put a clamp on the bleeding vessel. This will, of course, require a reopening of the wound or the making of a new one, all of which should be done as aseptically as the original operation.

Infection, primary or secondary, requires the free evacuation of pus and perfect drainage. The superficial wound may look beautiful, and yet there may be a large accumulation of pus under or over the fascia. This is not always accompanied by pain, so that the pulse and temperature, together with the general condition of the patient is often our only guide. The temperature will register 99° to 102° with perhaps a little chilly sensation; at times, sweating. The patient loses appetite, becomes restless, and has a general bad feeling. An infected ligature may be the cause, and then you will have to go deeper for the trouble. The wound, or sinus, as it may be, must be thoroughly irrigated twice daily, and the patient, as a rule, is no worse for this interruption in the convalescence. When a ligature is at fault, a sinus may persist for many months, until that foreign body escapes or is removed, when the wound closes in very short order. I had one case of appendicitis in which I had to make a counter-opening in the loin, and both this and the anterior sinus remained open for thirteen months, but upon the withdrawal of the ligature, both closed within a week. When a sinus first makes its appearance and the pus has been in close proximity to intestinal walls, the appearance, as well as odor, resemble very much that of feces, and often make the operator fear he has a fecal fistula to deal with. This odor and dark color of the discharge soon change to an odorless, clear pus. When this change takes place, the surgeon always feels like congratulating himself that he has no fistula of a rebellious nature to fight.

Dressings, when wounds are closed, should not be disturbed until you are ready to remove the sutures, providing the patient is very well. In cases where you have drainage by gauge in the vagina or abdominal wound, these should be looked after, often after the first thirty-six or forty-eight hours. A glass drain, if used, must be emptied every half hour, and each time should be turned to prevent granulations entering its caliber through the various little openings. The asepticism of the hands, dressings and instruments must be as perfect at each change of dressing as they were at the time of operation.

In removing sutures there is one little point to guard against, and that is, infecting the stitch hole by drawing through it some foreign matter on the surface of the suture. This can be avoided by not cutting the suture only in that part that is within the puncture. You can do this by pulling on the ligature and, at the same time, using the scissors open to push down on the skin and cut only when the blades are opposite the white suture material. In withdrawing the suture, draw it toward and not from the cut side. You thus avoid any traction on the scar and cause your patient much less pain.

### REMOTE INTESTINAL OBSTRUCTION.

Intestinal obstruction following abdominal and pelvic operations are by no means uncommon. Those that occur immediately after, or during the first week, are, as a rule, accompanied by septic peritonitis, and in most instances every plan of treatment is futile. In all instances it is best to open the abdomen at once, and after loosening any adhesions, thoroughly drain. The cases of obstruction that follow later, say within two years after the operation, are often very puzzling and insidious in their onset. It has been my misfortune to see two such cases within the last six weeks, and I will take the liberty to give a short sketch of the histories so as to bring out the importance of an early diagnosis.

CASE I.—Was in a young girl of about 16 years, who had been operated upon one year before by Dr. Mulligan for appendicitis. In the night of Friday, December 18, 1896, I was called to her and found her complaining of a sharp pain in the left side, over the region of the spleen. She had no fever and no local tenderness. The mother stated that she should have menstruated on the 15th, three days before, and thought that the delayed flow was the cause of her trouble. Her bowels had moved naturally that morning. The abdomen was not distended and she had not vomited. I ordered a turpentine enema and gave a hypodermic of morphia. At 8 A. M. on Saturday she was still in pain, with no fever or increased pulse, but had a ghastly expression. There were then no local signs of abdominal trouble. The enema had not been effectual and the mother took it upon herself to give a large dose of salts, followed by oil, and this was not successful. I gave a high enema and brought away some fecal matter, probably what was in the colon, but got no gas. While giving the enema the pulse became rapid. At noon she had her first temperature of 100°, pulse 120, and the face pinched and an anxious expression. It was at this visit that I had Dr. Mulligan see her. Even now she had no distention or local tenderness, yet it was impossible with every effort made to get a movement or gas from those bowels. We were then in hopes of stimulating her so as to be able to operate later in the afternoon, but she would not react in spite of the most active treatment and transfusion. She now began to vomit incessantly, and by 4 P. M. she was frightfully distended, began to have tenderness and died in a collapsed condition four hours later. No autopsy was permitted, but the diagnosis can hardly be doubted.

CASE II.—Mrs. P., age about 35, had been operated upon by our president one year before for dermoid cyst of the right ovary and cystic degeneration of the left. I was sent for on Wednesday, January 6, 1897, at 2 P. M., the patient complaining of a most severe abdominal pain. No fever; pulse, 80; bowels had moved the day before and also on that morning. I saw her twice more on that day and each time was compelled to give a hypodermic of morphia. At no time did she have fever, nor was the pulse over 80, but she had that same "abdominal expression," I would term it, that was present in Case I. The next morning I had Dr. Williams see the case with me. She had vomited some and the numerous enemas and cathartics ordered on the preceding evening were not effectual. She had no marked distention and no fever; her pulse, however, was very rapid, and she seemed in a collapsed condition. We nevertheless took the chance and sent her to the hospital, and after some stimulation had Dr. Little operate at nine o'clock that night. He found the obstruction caused by adhesion of the gut to the abdominal wound. The intestines were red and congested. The abdominal cavity contained some serum, but no pus. There was no doubt of beginning peritonitis. In one place, the gut was torn by loosening the adhesions and required a few sutures in its outer two coats. The patient reacted well and made a good recovery. She is now at her home, able to superintend her household.

Now, in neither of these cases were there the usual signs and symptoms at the onset of the trouble, and yet within twenty-four hours the one was so completely collapsed that operative measures could not be taken, and the other was saved only by a timely and systematic surgical procedure. If these cases teach you no lesson, they still impress me very strongly, and should I again find a patient that had the peritoneal cavity opened, and the same acute pain with vomiting later, and that peculiar expression described, I would advise immediate laparatomy.

When an abdomen has been opened, it is well to have the patient wear a good-fitting abdominal bandage of some strong material for at least one year. The question of allowing a corset is often met by a refusal from the surgeon. I can see no possible harm in its use, if it is comfortable to the individual case.

As for leaving home to complete a convalescence, I believe the patient should not be hurried into this. After leaving the hospital, they will get quite change enough at first by going into their own homes and not at once be compelled to rush into some fashionable hotel in the mountains or at the seaside. A change of that sort several months later, I think much better, and attended by much less excitement.

Note.—Since reading the above my attention has been called to a very good article on After-treatment in Abdominal Section in the *American Text-book of Gynecology.*—S. L. E.

83 North St. Paul Street.

